# A New Ultra-High-Strength AB83 Alloy by Combining Extrusion and Caliber Rolling

**DOI:** 10.3390/ma13030709

**Published:** 2020-02-05

**Authors:** Shuaiju Meng, Lishan Dong, Hui Yu, Lixin Huang, Haisheng Han, Weili Cheng, Jianhang Feng, Jingjing Wen, Zhongjie Li, Weimin Zhao

**Affiliations:** 1 School of Materials Science and Engineering, Hebei University of Technology, Tianjin 300130, China; shuaijumeng@163.com (S.M.);; 2 CITIC Dicastal Co., LTD, Qin Huangdao 066011, China; 3 Tianjin Key Laboratory of Materials Laminating Fabrication and Interfacial Controlling Technology, Tianjin 300130, China; 4 School of Materials Science and Engineering, Taiyuan University of Technology, Taiyuan 030024, China; 5 School of Materials Science and Engineering, Shanghai Jiao Tong University, Shanghai 200240, China

**Keywords:** Mg–Al–Bi alloy, caliber rolling, bimodal, mechanical properties

## Abstract

An exceptionally high-strength rare-earth-free Mg–8Al–3Bi (AB83) alloy was successfully fabricated via extrusion and caliber rolling. After three-pass caliber rolling, the homogenous microstructure of the as-extruded AB83 alloy was changed to a necklace-like bimodal structure consisting of ultra-fine dynamic recrystallized (DRXed) grains and microscale deformed grains. Additionally, both Mg_17_Al_12_ and Mg_3_Bi_2_ nanoprecipitates, undissolved microscale Mg_17_Al_12_, and Mg_3_Bi_2_ particles were dispersed in the matrix of caliber-rolled (CRed) AB83 alloy. The CRed AB83 sample demonstrated a slightly weakened basal texture, compared with that of the as-extruded sample. Consequently, CRed AB83 showed a tensile yield strength of 398 MPa, an ultimate tensile strength of 429 MPa, and an elongation of 11.8%. The superior mechanical properties of the caliber-rolled alloy were mainly originated from the combined effects of the necklace-like bimodal microstructure containing ultra-fine DRXed grains, the homogeneously distributed nanoprecipitates and microscale particles, as well as the slightly modified basal texture.

## 1. Introduction

Mg alloys are the lightest metallic structural materials available. Nowadays, there has been an increasing interest in them for lightweight applications to reduce pollution emissions and improve fuel efficiency. However, their commercial application is still very limited because of their poor mechanical performance and high processing cost compared with steel, Ti, and Al alloys [1]. Developing low-cost rare-earth free (RE-free) Mg alloys with high strength and good ductility is considered a prerequisite for their wider commercial adoption [1,2,3]. By incorporating grain refinement, precipitation hardening, and texture hardening, high strength has been achieved so far in Mg–Al- [4,5], Mg–Zn- [6,7,8], Mg–Ca- [9,10], and Mg–Sn- [11,12] based alloys. In recent years, several Mg–Bi-based alloys with attractive mechanical properties have been obtained [13,14,15,16]. Remennik et al. [14] fabricated a high ductile Mg–5Bi–1Ca wt.% alloy (all compositions quoted in the present study are in wt.% unless otherwise stated) having a high tensile elongation (EL) of over 40% as a result of rapid solidification and extrusion. Subsequently, a highly ductile Mg–1.32Bi–0.72Ca alloy having an EL of ~43% was successfully developed by single-step extrusion [17]. In addition, Somekawa et al. [13] produced a low-temperature (105–210 °C) extruded Mg–2.5Bi alloy with an ultra-high EL of 170%. However, these Mg–Bi-based alloys have low tensile yield strength (TYS), varying from 100 MPa to 205 MPa. Aiming to developing high-strength Mg–Bi-based alloys, we developed [15] a new as-extruded Mg–8Bi–1Al–1Zn (BAZ811) alloy, reaching a moderate TYS of 291 MPa. A further improvement on the strength of Mg–Bi-based alloys is still expected.

In recent years, a variety of process technologies has been developed to enhance the strength of Mg alloys, such as severe plastic deformation (SPD) processing technologies [18,19], low-temperature and slow-speed extrusion (LTSS-E) [20], and double extrusion (DE) [10,21]. For example, through SPD processes such as equal channel angular extrusion (ECAP) [19], high-ratio differential speed rolling (HRDSR) [22], accumulative roll bonding (ARB) [23], or multi-directional forging (MDF) [24] high-strength Mg–Al–Zn-based alloys, with the TYS ranging from 347 MPa to 480 MPa, have been successfully fabricated. We [20] also successfully developed the low-temperature slow-speed extruded (LTSS–Eed) AZ80 alloy with an extremely high TYS of 403 MPa, owing to the synthetic effects of grain refinement, precipitation strengthening, and texture strengthening. In addition, Naoko Ikeo et al. fabricated a dilute Mg–0.5Ca alloy with a high TYS of 400 MPa by DE [10]. However, these processing technologies have been difficult to commercialize due to low processing efficiency and limited size of the samples.

Consequently, caliber rolling was recently introduced as one of the modified rolling processes which could be used for mass production of metals with high precision and high strength. Lee et al. [25] fabricated a Ti–Al–V alloy with ultra-fine grains by caliber rolling. In spite of the great potential of grain refinement, there are just a few research groups working on caliber-rolled (CRed) Mg-based materials. The corresponding mechanical properties are summarized in Table 1 [26,27,28,29]. Somekawa et al. [26] found that the TYS of as-extruded Mg–Al, Mg–Zn, Mg–Ca, Mg–Sn, and Mg–Y binary alloys was significantly improved after 14-pass caliber rolling. Lee et al. [27] reported that a seven-pass CRed AZ31 alloy showed TYS, ultimate tensile strength (UTS), and EL of 298 MPa, 378 MPa, and 20.9%, respectively. Later, they also [28] fabricated an ZK60 alloy with an ultra-fine structure yielding a TYS of 364 MPa and an EL of 18%, using 6-pass caliber rolling. In addition, Mukai et al. [29] revealed a weakened effect of the basal texture and the enhancement of TYS when 18-pass caliber rolling was applied to the AZ31 alloy. All these results demonstrated the great potential of caliber rolling in improving the mechanical properties of Mg alloys. However, the number of caliber-rolling passes applied was too high, inevitably decreasing production efficiency and increasing costs.

Therefore, in this context, an Mg–Al–Bi-based alloy with good mechanical properties was successfully fabricated by combining hot extrusion and three-pass caliber rolling. Additionally, the microstructure of the CRed alloy was systematically investigated by comparing it with the as-extruded sample to reveal the strengthen mechanism.

## 2. Materials and Methods

**Figure 1:**
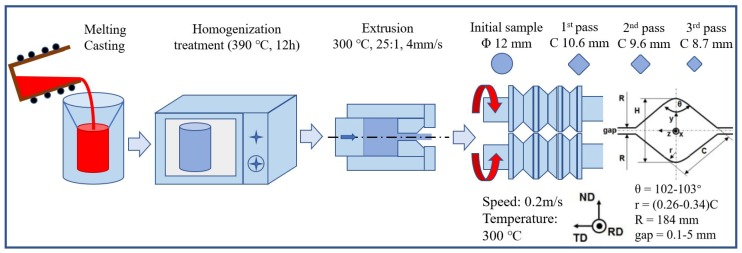
Schematic illustration of cast, homogenization, extrusion, and caliber rolling.

An Mg alloy with a designed composition of Mg–8Al–3Bi wt.% (denoted as AB83 hereafter) was fabricated by melting high-purity Mg, Al, and Bi, using an induction melting furnace under an inert atmosphere of mixed SF_6_ and CO_2_. As illustrated in Figure 1, after melting, the melt was poured into a permanent mold at 720 °C. Apart from Mg, 8.13% of Al, 2.92% of Bi, 0.004% of Zn, 0.0029% of Mn, 0.0005% of Fe, 0.0119% of Ni, 0.0317% of Ca, 0.0005% of Si, and 0.0025% of Ti were also detected using an optical emission spectrometer (OES, OBLF, Shanghai, China). Furthermore, the composition of the main element in the as-cast sample was confirmed as Mg–8.2Al–2.9Bi by inductively coupled plasma (ICP) analysis and appeared consistent with the designed composition. The homogenization treatment of the billet, with a diameter of 60 mm and a height of 150 mm, was conducted at 390 °C for 12 h. The extrusion process was carried out at 300 °C with an extrusion ratio of 25:1 at a ram speed of 4 mm/s. Then, the as-extruded bar, with a length of 50 mm and a diameter of 12 mm, was processed by a three-pass caliber rolling with grooves from 10.6 to 8.7 mm without lubrication at 300 °C. The rolling speed was 0.2 m/s, and the accumulated strains of the three-pass caliber rolling was ~40.2%. Both the extrusion and the caliber rolling of the AB83 alloy were performed in an ambient atmosphere.

Metallographic samples for microstructure characterization were cut from the as-extruded and CRed AB83 alloy along the extrusion direction (ED) and the rolling direction (RD), respectively. Microstructure characterization was carried out using an Olympus BX51M optical microscope (OM, OLYMPUS, Tianjin, China), a JEOL JSM-7000F scanning electron microscope (SEM, JEOL, Tianjin, China) equipped with an energy dispersive spectrometer (EDS), and a transmission electron microscope (TEM) equipped with an energy-dispersive X-ray spectroscopy (EDX). In order to figure out the phase in the specimens, X-ray diffraction (XRD) analysis was conducted using Bruker D8 Focus (Tianjin, China). A solution of 4.2 g of picric acid, 70 mL of ethanol, 10 mL of acetic acid, and 10 mL of distilled water was used to etch the specimens for OM and SEM observation. Thin disc samples for the TEM observations, with a diameter of 3 mm, were mechanically polished to a thickness less than 200 μm, followed by ion milling using a GATAN 691 Precision Ion Polishing System (Technoorg Linda, Shenyang, China). For EBSD examination, the surface of the specimens was polished by colloidal silica for 30 min. HKL Channel 5 analysis software (OXFORD INSTRUMENTS, Shenyang, China) was used to analyse the EBSD data.

In addition, dog-bone-shaped specimens with gage dimension of Φ4 mm × 18 mm were used for the tensile tests. The tensile tests were conducted using an electro-universal mechanical testing machine (SUNS-UTM5105X, SHENZHEN SUNS TECHNOLOGY STOCK CO., LTD., Tianjin, China) at room temperature, with an initial strain rate of 1 × 10^−3^ s^−1^ along the ED or the RD. All samples were tested at least three times to confirm the repeatability of the mechanical properties. After the tensile tests, the fracture morphologies of the caliber-rolled sample were characterized by SEM and compared with those of the as-extruded sample. 

## 3. Results and Discussion

### 3.1. Microstructure Characteristics

**Figure 2:**
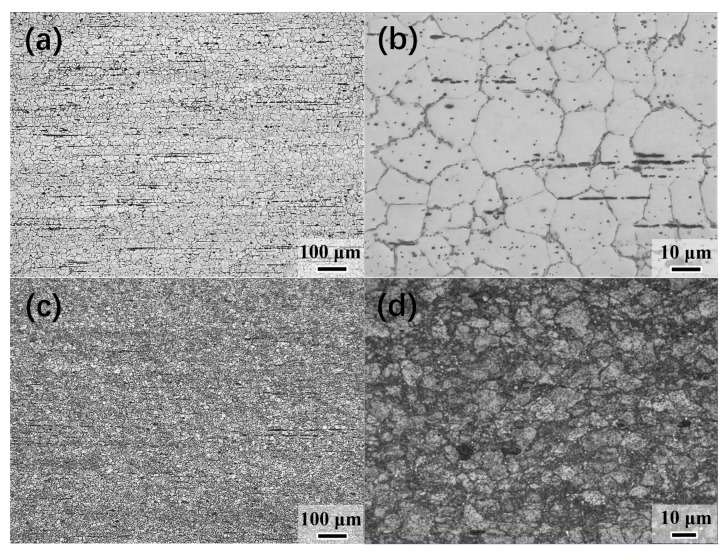
Optical microscope (OM) images of (**a**,**b**) as-extruded and (**c**,**d**) caliber-rolled AB83 alloy.

Figure 2 indicates the OM microstructure of the as-extruded (Figure 2a,b) and caliber-rolled (Figure 2c,d) AB83 alloy. The as-extruded AB83 alloy exhibited a homogeneous microstructure with an average grain size (AGS) of ~16 μm. After three-pass caliber rolling, the CRed sample exhibited a typical bimodal microstructure, which consisted of deformed grains from the as-extruded state and fine dynamic recrystallized (DRXed) grains formed along the boundaries of the deformed grains. The volume fraction of DRXed grains was ~45% (Figure 2c,d). The formation of a necklace-like structure in the CRed sample suggested that discontinuous DRX (DDRX) occurred during the three-pass caliber rolling process at 300 °C [30]. In addition, both nanoprecipitates and microscale second particles were also observed along the ED in the as-extruded sample. Also, most of the microscale second phases were further fractured and redistributed during the subsequent caliber rolling progress.

**Figure 3:**
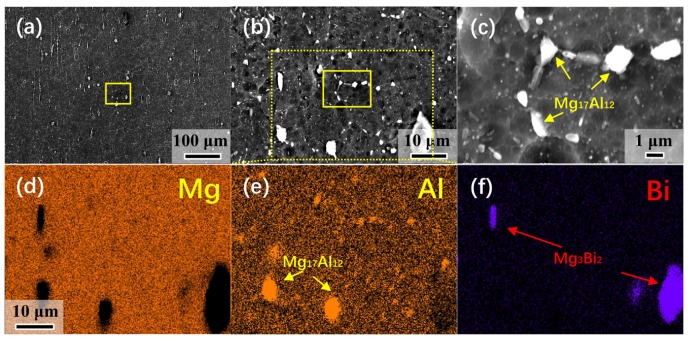
(**a**–**c**) Scanning electron microscopy (SEM) micrographs and (**d**–**f**) energy dispersive spectrometry (EDS) of as-rolled AB83 alloy. Arrows indicate Mg_3_Bi_2_ and Mg_17_Al_12_ second particles.

**Figure 4:**
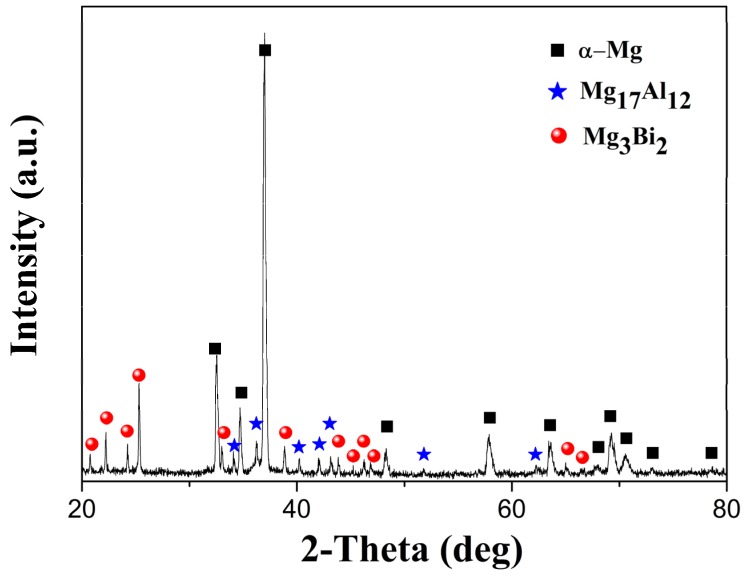
X-ray diffraction (XRD) pattern of caliber-rolled AB83 alloy.

Figure 3 presents the SEM micrographs (Figure 3a–c) and EDS (Figure 3d–f) of the CRed AB83 sample, with clear second phases. As shown in Figure 3c, both microscale and nanoscale second-phase particles were detected. Furthermore, the EDS results (Figure 3d–f) confirmed that these second phases included both Mg–Bi and Mg–Al phases. Combined with the XRD analysis results of CRed AB83, as demonstrated in Figure 4, these second phases were confirmed as Mg_3_Bi_2_ and Mg_17_Al_12_, respectively. This result was different from that obtained for Mg–8Bi–1Al–1Zn alloy [15], in which only the Mg_3_Bi_2_ phase was detected. This was mainly due to the higher content of Al in AB83 and the lower homogenization treatment temperature used in this study compared with those used for BAZ811 alloy [15].

**Figure 5:**
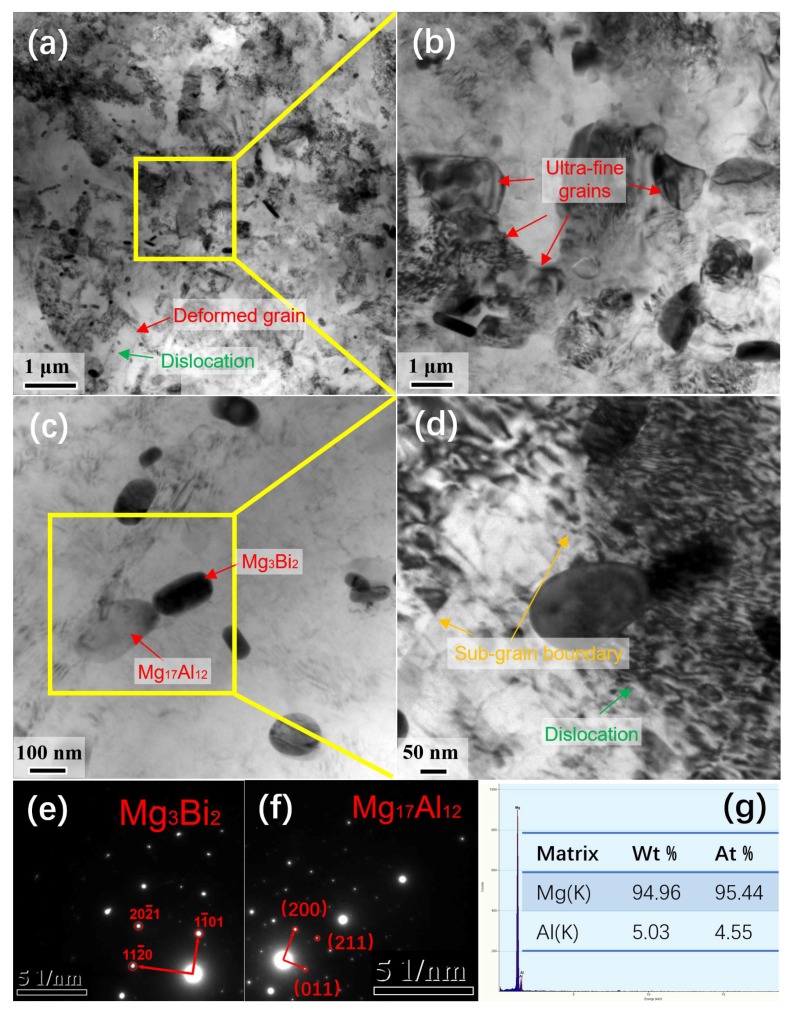
Bright-field transmission electron microscope (TEM) images (**a**–**d**), diffraction patterns of the selected areas (**e**–**g**) energy-dispersive X-ray spectroscopy (EDX) results for the matrix of the caliber-rolled AB83 alloy.

In order to further analyze the microstructure of the CRed AB83 alloy, a TEM experiment was carried out. Bright-field TEM images are displayed in Figure 5. As shown in Figure 5a,b, both microscale deformed grains and ultra-fine DRXed grains were detected. In addition, nanoprecipitates were also detected homogeneously dispersed in the matrix (Figure 5a,c,d). Based on the selected areas of the electron diffraction results showed in Figure 5e,f, these nanoscale precipitation particles were confirmed to be Mg_3_Bi_2_ (hcp, a = 0.4666 nm, c = 0.7401 nm) and Mg_17_Al_12_ (bcc, a = 1.056 nm), respectively. According to the EDX results of the matrix in Figure 5g, there was an amount of Al element homogenized in the matrix. Moreover, as indicted by the green arrows in Figure 5a,d, residual dislocation lines could be detected in the deformed grains, and nanoprecipitate particles were found near the residual dislocation lines. Residual dislocations with high density, which partially transformed into subgrains, were readily detected, as indicated by the yellow arrows in Figure 5d. It was hypothesized that these sub-grains and the dislocations might have contributed to the potential nuclei of recrystallization. As shown in Figure 5a,b, some nuclei had grown to grains, thus filling the original grains, according to continuous DRX (CDRX) [31]. Furthermore, some of the precipitates, including both Mg_3_Bi_2_ and Mg_17_Al_12_ particles, were distributed at both the DRXed grain boundaries (Figure 5a,b) and in their interior (Figure 5c), suggesting that ultra-fine grains could be achieved by the pinning effect on the grain boundary movement in the present caliber-rolling conditions. As a result, the ultra-fine grain size of AB83 alloy (~0.9 μm) was achieved through both DDRX, along grain boundaries, and CDRX, in the interion of the grain, and was effectively pinned by nanoprecipitates. Consistently with previous research, ultra-fine grains were also obtained in LTSS–Eed Mg–8Al–4Sn–2Zn (~0.8 μm) [4], DEed Mg–0.5Ca [10], and ARBed AZ91 [24]. Accordingly, submicron grains and sub-grains, residual dislocations, solute atoms, as well as micro-scale and nanoscale second-phase particles, all present in the bimodal microstructure, should contribute to a high strength of the CRed AB83 alloy.

**Figure 6:**
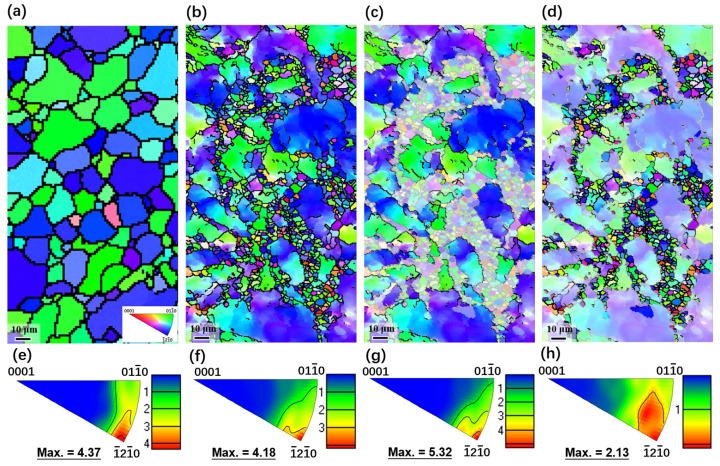
(**a**–**d**) Inverse pole figure (IPF) maps and (**e**–**h**) IPFs of (**a**,**e**) as-extruded and (**b**–**d**,**f**–**h**) caliber-rolled (Cred) AB83 alloy; (**b**,**f**) overall, (**c**,**g**) undynamic recrystallized (unDRXed) and (**d**,**h**) DRXed regions of the CRed AB83 alloy.

Figure 6 shows the EBSD analysis results for both as-extruded and CRed AB83 alloys. The as-extruded AB83 alloy exhibited a uniform microstructure with an AGS of ~15 μm (Figure 6a). After three-pass caliber rolling, both the deformed coarse grains, with the volume fraction of ~65%, and the ultra-fine grains, with a diameter ~1 μm, could clearly be observed along the boundaries of the deformed grains in the inverse pole figure (IPF) maps (Figure 6b–d). The results from EBSD were in line with our OM and TEM observations. In addition, both the as-extruded and the CRed AB83 alloys exhibited basal fiber texture (Figure 6e–h) with the {0001} plane and the <−12–10> direction of the grains mainly oriented parallel to the ED and the RD in as-extruded and CRed samples, respectively. Specifically, the basal texture intensity of the CRed samples was 4.18 multiples uniform density (mud.), which was a little lower than that of the AB83 alloy not subjected to caliber rolling (4.37 mud.). The basal texture weakening effect of the caliber rolling process on the extruded Mg alloy, which was mainly caused by shear deformation, was also revealed by previous research on AZ31 by Mukai et al. [29]. It should be noted that the deformed grains in the CRed sample demonstrated strong <−12–10> // RD texture with a maximum intensity of 5.32 mud., while more randomized texture (maximum intensity, 2.13 mud.) was generated in the ultra-fine DRXed grains, as indicated in Figure 6g,h, respectively. This phenomenon was consistent with previous results of the texture transition during DRX [15,32]. As a result, in this study, a bulk Mg alloy having a necklace-like structure and a modified basal texture was successfully fabricated by combining two simple thermomechanical processes. The necklace bimodal microstructure, which was reported to be beneficial to both strength and ductility, was also observed in the hard plate-rolled (HPRed) Mg–9Al–1Zn alloy [33].

### 3.2. Mechanical Properties

**Figure 7:**
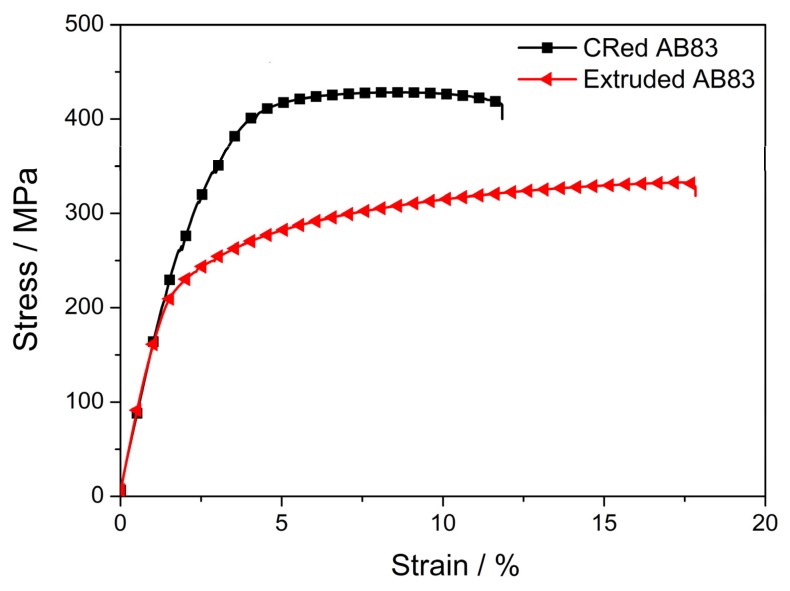
Typical tensile engineering stress–strain curves of the CRed AB83 alloy and of the as-extruded AB83 alloy.

The mechanical properties obtained from the tensile tests of both CRed and as-extruded AB83 alloys are given in Table 2, and the typical tensile engineering stress–strain curves are shown in Figure 7. The as-extruded AB83 alloy was of medium strength, with YS and (UTS) of 242 MPa and 332 MPa, respectively. After three-pass caliber rolling, the CRed AB83 alloy demonstrated an extraordinarily high strength, with YS of 398 MPa and UTS of 428 MPa, both significantly higher than those of the as-extruded sample. Additionally, the TYS of the CRed AB83 alloy was higher than those of the newly developed Mg–6Bi [16], BAZ811 [15], and Mg–8Al–4Sn–2Zn [4] alloys. Furthermore, the YS of the CRed AB83 alloy was higher than those of the previously reported 12-pass ECAPed Mg–3.7Al–1.8Ca–0.4Mn [19], DEed Mg–0.5Ca [10], 6-pass CRed ZK60 [28], HRDSRed AZ91 [23], 20-pass MDFed AZ61 [22], and RE-containing Mg–8Gd–3Y–1Zn wrought alloy [34]. The high strength of the CRed AB83 alloy was mainly due to the synthetic effect of the following factors: grain refinement of the ultra-fine DRXed grains, precipitation strengthening resulting from the fine Mg_3_Bi_2_ and Mg_17_Al_12_ precipitates, dispersion strengthening from the undissolved Mg_3_Bi_2_ and Mg_17_Al_12_ particles, and the solid-solution strengthening promoted by dissolved Al.

**Figure 8:**
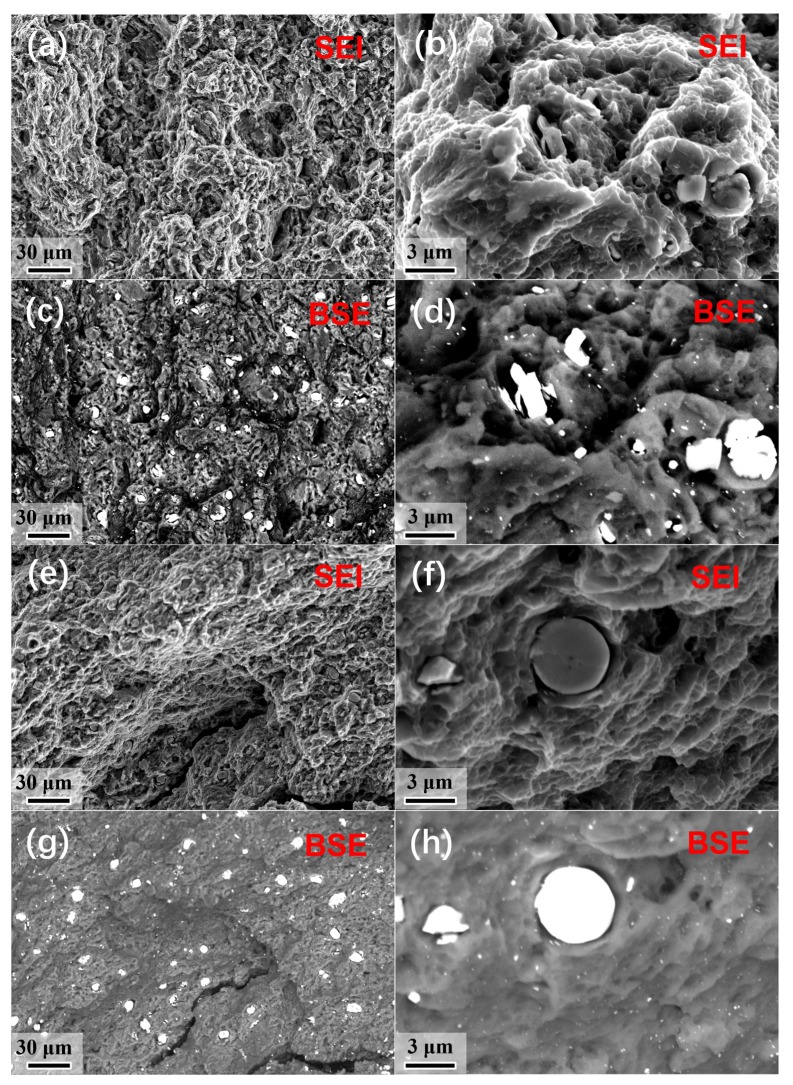
SEM fracture morphology of (**a**–**d**) as-extruded and (**e**–**h**) CRed AB83 alloy after failure during the tensile test.

On the other hand, the CRed AB83 sample, characterized by a much higher strength, demonstrated a lower EL of 11.8% compared to the as-extruded sample (EL of 17.6%). This could be explained by the morphology of the fracture surface after the tensile test. As demonstrated in Figure 8, the fracture morphology of both as-extruded and CRed samples showed a ductile characteristic with plenty of dimples on the surface. In addition, both microscale and nanoscale second-phase particles could be observed on the fracture surface of the two samples (Figure 8d,h). This observation is in line with the above microstructure characterization results. However, the surface morphology of the as-extruded sample was more uniform than that of the CRed sample. This difference could be attributed to the homogenized and bimodal microstructure of the as-extruded and CRed samples, respectively. As the strain hardening ability of the ultra-fine grain area was lower than that of the micro-scale grain area [15,35,36], the inhomogeneous deformation of the ultra-fine grains and microscale grains in the CRed sample initiated a fracture, as shown in Figure 8e,g.

**Figure 9:**
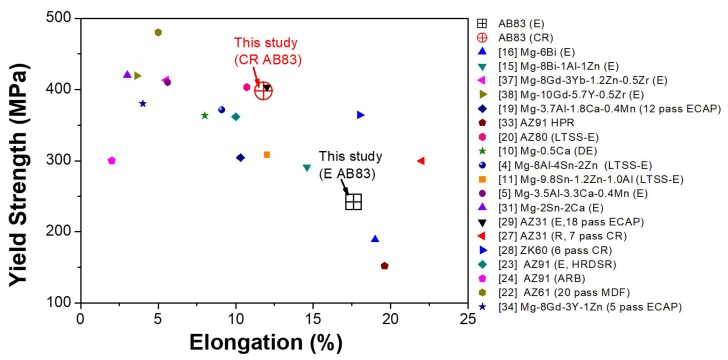
Comparison of UTS vs. EL of CRed AB83 with those of previously reported wrought Mg alloys [4,5,10,11,15,16,19,20,22,23,24,27,28,29,31,33,34,37,38] (E: extrusion; CR: caliber rolling; ECAP: equal channel angular pressing; HPR: hard-plate rolling; LTSS–E: low-temperature slow-speed extrusion; DE: double extrusion; HRDSR: high-ratio differential speed rolling; ARB: accumulative roll bonding; MDF: multidirectional forging).

As shown in Figure 9, even though the EL of CRed AB83 was lower than that of as-extruded AB83, the ductility of the first (~11.8%) was one of the highest recorded for ultra-high strength Mg alloys [4,5,10,11,15,16,19,20,22,23,24,27,28,29,31,33,34,37,38]. Compared with the newly developed high-strength Mg–3.5Al–3.3Ca–0.4Mn [5] and Mg–2Sn–2Ca [31] alloys, which were reported to have the two highest YS among RE-free Mg alloys, the CRed AB83 alloy showed a slightly lower YS but a much greater EL. The good comprehensive mechanical performance of the CRed AZ80 alloy was mainly due to its necklace bimodal structure. The development of a bimodal microstructure has been proposed as a good way to produce metal materials having both high strength and good ductility. In a bimodal microstructure, the micro-scale grains guarantee strain hardening by providing more space to accommodate dislocations, while the ultra-fine grains allow for a higher strength [39,40,41]. This strategy was recently verified using a hard-plate rolling (HPR)-processed AZ91 alloy with a necklace bimodal structure [33]. In addition, the necklace bimodal microstructure with more random basal texture benefits from the glide dislocation on basal planes, which makes the CRed AB83 alloy more ductile [42,43].

## 4. Conclusions

In summary, we successfully developed an extraordinarily high-strength RE-free AB83 alloy by combining extrusion and three-pass caliber rolling. The CRed sample with a necklace bimodal structure exhibited a high YS of ~398 MPa and a good ductility of ~11.8%. The attractive performance was mainly attributed to the synthetic effect of a necklace bimodal microstructure containing ultrafine grains. The profuse Mg_3_Bi_2_ and Mg_17_Al_12_ precipitates, the residual dislocations, and the dissolved Al atoms also enhanced the strength and the modified texture that potentiated the ductility. In addition, the evidence for both DDRX and CDRX in CRed AB83 alloy were presented. Overall, the RE-free cheap alloying elements and the simple combination of two commercially available processing technologies, extrusion and caliber rolling, are expected to inspire both new alloying strategies and processing methods, towards the large-scale production of high-performance Mg alloys.

## Figures and Tables

**Table 1:** Mechanical performance of caliber-rolled (CRed) Mg alloys compared with the corresponding as-extruded samples [26,27,28,29]. TYS: tensile yield strength, UTS: ultimate tensile strength, El: tensile elongation.

Alloys	Process	TYS (MPa)	UTS (MPa)	El. (%)	Ref.
Mg–0.33Al	E ^1^, 4:1 + 14 pass CR ^2^, 300 °C	311	-	16.5	[26]
Mg–0.33Al	E, 4:1 + E, 19:1, 185 °C	205	-	16.6	[26]
Mg–0.49Ca	E, 4:1 + 4 pass CR, 300 °C	282	-	13.7	[26]
Mg–0.49Ca	E, 4:1 + E, 19:1, 225 °C	318	-	4.4	[26]
Mg–1.45Sn	E, 4:1 + 14 pass CR, 300 °C	243	-	27.7	[26]
Mg–1.45Sn	E, 4:1 + E, 19:1, 175 °C	11.6	-	5.9	[26]
Mg–1.09Y	E, 4:1+ 6 pass CR, 400 °C	271	-	15.8	[26]
Mg–1.09Y	E, 4:1 + E, 19:1, 290 °C	289	-	14.7	[26]
Mg–0.8Zn	E, 4:1 + 14 pass CR, 300 °C	330	-	16.0	[26]
Mg–0.8Zn	E, 4:1 + E, 19:1, 185 °C	190	-	23.1	[26]
AZ31	R ^3^	155	290	16	[27]
AZ31	R+ 1 pass CR, 400 °C	145	270	22	[27]
AZ31	R+ 3 pass CR, 400 °C	200	310	23	[27]
AZ31	R+ 5 pass CR, 400 °C	240	320	24	[27]
AZ31	R+ 7 pass CR, 400 °C	300	370	22	[27]
ZK60	6 pass CR, 400 °C	364	389	18	[28]
ZK60	E	266	321	15	[28]
AZ31	E, 400 °C, 16:1	230	280	13	[29]
AZ31	E + 15 pass CR, 200 °C	345	358	13.5	[29]
AZ31	E + 18 pass CR, 200 °C	405	410	12	[29]

^1^ E: Extrusion; ^2^ CR: caliber rolling; ^3^ R: rolling.

**Table 2:** Mechanical performance of CRed Mg alloys compared with corresponding as-extruded sample [26,27,28,29].

Alloys	Process Parameters	TYS (MPa)	UTS (MPa)	EL (%)
AZ80	E ^1^, 300 °C, 4 mm/s	242 ± 3	332 ± 4	17.6 ± 2
AZ80	E + 3 pass CR ^2^, 300 °C	398 ± 2	429 ± 4	11.8 ± 2

^1^ E: Extrusion; ^2^ CR: caliber rolling.
